# Lower Retinal Arteriolar Density Is Associated With Higher Cerebral Small Vessel Disease Burden: An Optical Coherence Tomography Angiography Study

**DOI:** 10.1002/brb3.70342

**Published:** 2025-02-17

**Authors:** Le Cao, Hang Wang, Jinkui Hao, William Robert Kwapong, Zhouwei Xiong, Ruilin Wang, Chen Ye, Yitian Zhao, Bo Wu, Wendan Tao

**Affiliations:** ^1^ Department of Neurology, West China Hospital Sichuan University Chengdu China; ^2^ Cixi Institute of Biomedical Engineering Ningbo Institute of Materials Technology and Engineering, Chinese Academy of Sciences Ningbo PR China; ^3^ Department of Ophthalmology, West China Hospital Sichuan University Chengdu China

**Keywords:** arterioles, optical coherence tomography angiography, perivascular spaces, venules, white matter hyperintensities

## Abstract

**Introduction:**

It is suggested that cerebral small vessel disease (SVD) plays a role in strokes and dementia. Retinal microvasculature imaged by optical coherence tomography angiography (OCTA) is suggested to be associated with cerebral microvessels. We measured the density of the retinal arterioles and venules on the superficial vascular complex (SVC) of OCTA images and investigated associations with SVD markers in older adults.

**Methods:**

Participants underwent cerebral magnetic resonance imaging and OCTA imaging. An external algorithm (OCTA‐Net) was used to segment the retinal vessels into arterioles and venules. SVD indicators [lacunes, white matter hyperintensity (WMH), perivascular spaces (PVS) and cerebral microbleeds (CMBs)] were determined according to the STandards for ReportIng Vascular changes on nEuroimaging (STRIVE)

**Results:**

246 older adults (mean age = 58.84 ± 7.00 years; 38.21 % males) were included in our data analysis. After adjusting for covariates, lower retinal arteriole densities correlated with higher periventricular WMH (*p* = 0.025) and PVS in the basal ganglia (*p* = 0.027). Lower retinal venule density correlated with higher deep WMH burden (*p* = 0.014). Lower arteriolar density was associated with increased SVD burden (*p* = 0.035). Arteriolar complex branching was associated with periventricular WMH (*p* = 0.020) while venular complex branching was associated with deep WMH (*p* = 0.041).

**Conclusion:**

Retinal vascular changes may reflect cerebral vascular changes as evidenced by OCTA‐derived metrics.

## Graphical abstract

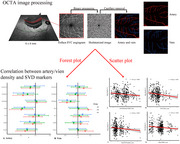

## 1 Introduction

Cerebral small vessel disease (SVD) has unclear mechanisms and treatment options are limited. As part of the pathophysiology of SVD, impaired vasodilation, subtle leakage of the blood‐brain barrier, and stiffening of vessels are all thought to occur at the level of the perforating microvasculature of the brain (Duering et al. 2023). SVD structural brain imaging features include lacunes, white matter hyperintensities (WMH), perivascular spaces (PVS) and cerebral microbleeds (Duering et al. 2023). Magnetic resonance imaging (MRI) can reveal structural brain imaging features of SVD indicating cerebral vascular dysfunction.

Anatomically and physiologically, the retinal microvasculature shares many features with similar sized vessels in the brain (Patton et al. 2005). We may be able to draw useful conclusions about the microvascular health of the brain by examining the retina's microvasculature based on noninvasive imaging captured in fundus photography and optical coherence tomography angiography (OCTA) (Kashani et al. 2017). Microvascular changes might even be detected by OCTA before they are visible by brain imaging methods as shown in previous studies (Liu et al. 2023; Lopez‐Cuenca et al. 2021; Pujari et al. 2021; Zhang et al. 2023).

Compared to controls, accumulating studies using fundus photography have shown that patients with stroke, dementia and other neurological disorders have wider venules, smaller arterioles, and a diminished fractal dimension of the microvascular network (Cheung et al. 2017; Kashani et al. 2021). Moreover, retinal microvascular abnormalities (such as focal arteriolar narrowing and arteriovenous nicking) observed on fundus imaging are associated with incident lacunar stroke during follow‐up, and retinopathy (such as microaneurysms, hemorrhages, and exudates) is associated with dementia and stroke (Kashani et al. 2021; McGrory et al. 2017).

SVD markers in the brain have also been associated with fundus imaging findings. The PVS volume increased with decreasing fractal dimension and the retinal vessel branching coefficients increased with periventricular WMH (Ballerini et al. 2020; Doubal et al. 2010).

OCTA is an imaging modality that noninvasively and quickly demonstrates the retinal microvasculature with high resolution (Kashani et al. 2017). Moreover, this technique provides detailed information about the retinal microvascular network in different layers. According to accumulating studies, patients with Alzheimer's disease (AD) and preclinical AD had changes in perfusion density and vessels on OCTA (Alber et al. 2020; Zhang et al. 2020), but sparse data prevented conclusions about OCTA findings in SVD and whether vessel density decreased with worse WMH (Zhang et al. 2020). Our previous reports showed that retinal vascular metrics (through semiautomated software) are associated with SVD markers in older adults (Tao et al. 2022); we showed that increased retinal superficial vascular complex (SVC) tortuosity is associated with increased deep WMH in older adults. Besides there is increasing evidence that the superficial vessels of the retina imaged and measured on OCTA are associated with SVD parameters such as lacunes, CMB and WMH.

Through semiautomated computer software, retinal microvascular parameters such as retinal arterioles and venules can now be measured in OCTA images. The retinal arterioles and venules have different functions, which have been detailed in previous fundus photography reports. In older adults, no studies have reported how retinal arterioles and venules measured using OCTA correlate with SVD metrics. However, to move toward using retinal arterioles and venules as biomarkers for SVD in the clinical setting, it is worthwhile to investigate this.

In this cross‐sectional study, we investigated the association between OCTA retinal vascular metrics (retinal arterioles and venules) with neuroimaging biomarkers of SVD. In the context of aging‐related cerebral disorders, our findings may shed significant light on microvascular changes that play a key role in underlying pathophysiology.

## 2 Methods

### 2.1 Study Population

We recruited volunteers at the West China Hospital who had no neurological disorders. Study participants were recruited from April 2021 to September 2023. The Sichuan University West China Hospital Institutional Review Board approved this study (Ethics number: 2020–104). All procedures were conducted according to the Helsinki Declaration 1975 (revised in 1983).

We included the following criteria: (1) age ≥ 50 years; (2) has the ability to read and understand Chinese Mandarin; (3) has the ability to cooperate with MR imaging and OCTA examinations.

We excluded the following: (1) history of clinically diagnosed AD; (2) history of cerebral stroke; (3) history of cerebral disorders such as Parkinson's disease, brain tumors, and epilepsy. Ophthalmological exclusion criteria were as follows: history of ocular surgery or ocular diseases such as age‐related macular degeneration, severe cataract, severe glaucoma, and macular and relevant opacities of the optic media.

Using questionnaires, we assessed demographics (age, gender, ethnicity, education, and smoking status) and medication usage for high blood pressure, high cholesterol, or diabetes mellitus. Nonsmokers and smokers were categorized based on their smoking status. Those who never smoked were classified as nonsmokers, and those who smoked regularly or had at least 100 cigarettes were classified as smokers. Hypertension was defined as self‐reported or use of antihypertensive medication or measured systolic blood pressure > 140 mm Hg and/or diastolic blood pressure > 90 mm Hg. A mercury sphygmomanometer was used to measure systolic and diastolic blood pressure, and their mean was calculated. Diabetes mellitus was identified as a self‐described diabetes history, and anti‐diabetic medication. Hyperlipidemia was defined as a self‐reported dyslipidemia diagnosis and dyslipidemia medication use.

### 2.2 MR Imaging

The MRI examinations were conducted using a standard 3T scanner (Siemens Skyra) equipped with a 32‐channel head coil in Wuhou Health Management Center of West China Hospital.

Cerebral small vessel disease MRI markers, including lacunes, white matter hyperintensity (WMH), cerebral microbleeds (CMBs) and perivascular spaces (PVS) were assessed according to the STandards for ReportIng Vascular changes on nEuroimaging (STRIVE) consensus criteria (Duering et al. 2023). Lacunes were rounded or ovoid lesions involving the subcortical regions, 3–15 mm in diameter, of CSF signal intensity on T2WI and FLAIR, generally with a hyperintense rim on FLAIR and no increased signal on DWI. WMH severity was evaluated on FLAIR using the Fazekas scale (Fazekas et al. 1987) and was rated (0–3) separately for deep and periventricular regions of the brain, with the sum of the scores representing the total WMH burden. CMBs were homogenous rounded hypointense lesions on susceptibility‐weighted imaging (SWI) with a diameter of 2–10 mm. PVS was defined as small (< 3 mm) round or linear hyperintense lesions on T2WI in the basal ganglia (BG) or centrum semiovale (CS) and was rated as 0–4 on a validated semiquantitative scale (Doubal et al. 2010). Ordinary score (0‐4) was established to reflect the total burden of SVD (Staals et al. 2014).

A rater (YC) blinded to the clinical information and OCT/OCTA data evaluated the MR images for all participants. A second rater (BW) rated a random sample of 50 participants to evaluate interrater agreement for presence of microbleeds (kappa 0.93, *p* < 0.001), the severity of WMH (kappa 0.91, *p* < 0.001), the presence of lacunes (kappa 0.92, *p* < 0.001), PVS in centrum semiovale (CSO) (kappa 0.88, *p* < 0.001) and in BG (kappa 0.91, *p* < 0.001).

### 2.3 Retinal Imaging With Swept Source Optical Coherence Tomography (SS‐OCT)

SS‐OCT (VG200S; SVision Imaging, Henan, China; version 2.0.106) was used for all retinal imaging. Our previous reports (Cao et al. 2024; Wang et al. 2022) detail the specifications of the SS‐OCT tool. Both eyes were imaged for all participants.

Fundus images were obtained with the OCTA tool. In a 6 mm area centered on the fovea, *en face* angiogram of the superficial vascular complex (SVC) was generated by automatic segmentation. This microvascular plexus includes vessels from 5 m above the inner limiting membrane (ILM) to lower two‐thirds of the inner plexiform layer (GCIPL).

Our data followed the OSCAR‐IB quality criteria (Tewarie et al. 2012) and APOSTEL recommendation (Aytulun et al. 2021). We excluded images with signal quality < 7, motion artifacts, and ophthalmic disorders like age‐related macular degeneration, diabetic retinopathy, moderate to severe glaucoma and severe cataracts. Whenever a participant presented with any of these disorders in one eye, the other eye was used; if the participant presented with the disorders in both eyes, the participant was excluded.

### 2.4 Vessel Density and Vessel Branching Complexity

Using a binary map of the vessels, the percentage of vessel area (white pixels to black background) occupying the image was calculated. By using OCTA‐Net, a preprocessing step was performed on the vessel structures before creating the binary map. Larger VD values reflect greater density.

This method quantifies the complexity of vascular patterns in retinal images by using fractal dimensions (FD), which are single values that measure how sparse or complex they are. To quantify the complexity of the vessel branching pattern, box counting was used. The unitless values reflect complexity, with larger numbers reflecting greater complexity.

### 2.5 Quantification of Arterioles and Venules

**FIGURE 1:**
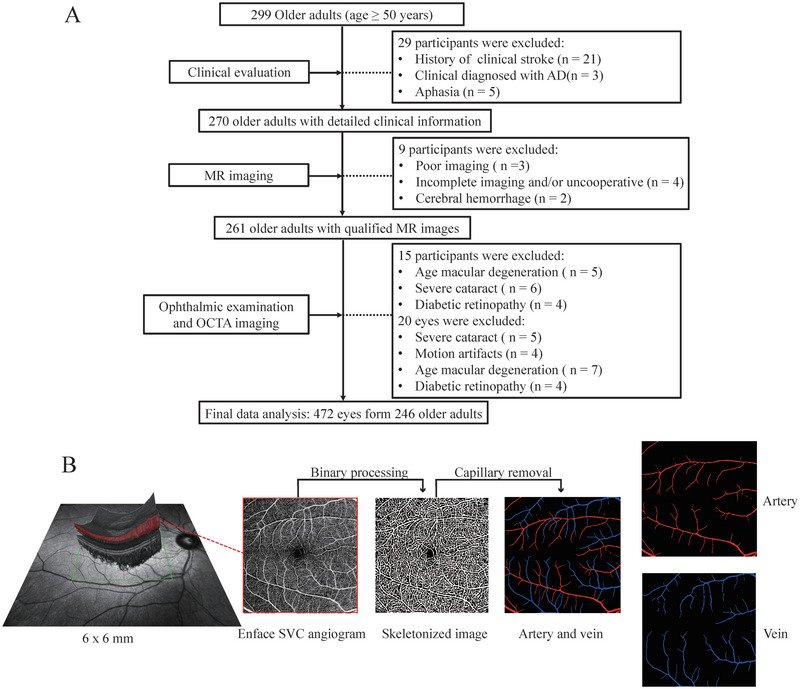
**Flow chart of enrollment of participant and SVC angiogram processing**. (A) Flow chart displaying the selection of participants. (B) SVC angiogram was processed to generate binary images and based on the final binary images; a skeletonized image was created. After imaging processing, capillaries were extracted from the image to detect arterioles and venules.

Full details on the classification of arterioles and venules in the SVC angiogram have been published (Cao et al. 2024; Xie et al. 2020). In brief, fundus imaging was performed and an ophthalmologist (Dr. Wang Ruilin) indicated the retinal arterioles and venules on the fundus image. The retinal arterioles and venules were annotated on the OCTA image while Gaussian process regression was applied to classify the arterioles and venules in the SVC angiogram via a rigid registration algorithm while small vessels were extracted (Figure 1). VD and FD were used to assess the arterioles and venules.

### 2.6 Power Analysis

Prior sample size estimation was not conducted because of limited previous reports. Post‐hoc power analysis (PASS 2021) was performed. The power for the association between arteriolar density and periventricular WMH, PVS in the basal ganglia or SVD burden was 0.76, 0.89 and 0.54 respectively. The power for the association between venular density and deep WMH was 0.89.

### 2.7 Statistical Analysis

Mean ± standard deviation (SD), medians and interquartile ranges (IQR) or frequencies and percentages (%) were used to present the characteristics of participants. The distribution of SVD markers was showed as bar plot.

All analyses were performed per participant and eye (left and right eye) as eye asymmetry may affect data. When analysis for per person, generalized estimating equations (GEEs) with exchangeable working correlation matric considering the intereye correlation, were used. As for analysis in per eyes, linear regressions were conducted to explore the association between SVD markers and OCTA metrics. Both raw analyses and adjusted analyses were conducted; the covariates in adjusted analyses for GEEs and linear regressions were age, sex, hypertension, diabetes mellitus, hyperlipidemia, smoking status.

Model estimates for GEEs and linear regressions were shown as standardized regression coefficient (*r*) with confident interval, using forest plot. Significant correlations were also showed as added variable plot; the axis of added variable plots were the residuals when these variables were regressed on the covariates. All analyses and plotting were conducted in R version 4.3.1. *p* values less than 0.05 were considered statistically significant.

## 3 Results

Figure 1 shows the recruitment of our study participants between April 2021 and September 2023. Our final data analysis included 246 older adults (mean age = 58.84 ± 7.00 years; 38.21 % males). Out of the 246 older adults, 57 (23.12 %) had hypertension, 23 (9.35 %) had diabetes mellitus and 71 (28.91 %) had hyperlipidemia. 234 right eyes and 238 left eyes were used in our data analysis. Table 1 displays the demographic, retinal, and SVD parameters of our study participants while Figure S1 shows SVD markers for each level.

**TABLE 1:** Characteristics of our study population.

	ALL
Patient, *n*	246
Eyes, *n*	472
OD, *n*	234
OS, *n*	238
Male, *n*	94 (38.21%)
Age, years	58.84 ± 7.00
Hypertension, *n*	57 (23.12%)
Diabetes, *n*	23 (9.35%)
Dyslipidemia, *n*	71 (28.91%)
Smoking, *n*	26 (10.57%)
Drinking, *n*	31 (12.60%)
SVD markers	
CMB, *n*	37 (15.16%)
Lacunes, *n*	15 (6.10%)
PWMH	1 [0–1]
DWMH	1 [1–1]
PVS‐BG	1 [1–1]
PVS‐CS	1 [1–2]
SVD burden	0 [0–1]
OCTA metrics	
Arteriole, density (%)	5.47 ± 0.62
Venule, density (%)	5.51 ± 0.71

OD: right eye; OS: left eye; CMB: cerebral microbleeds; PWMH: periventricular white matter hyperintensity; DWMH: deep white matter hyperintensity; PVS‐BG: perivascular spaces in basal ganglia; PVS‐CS: periventricular spaces in centrum semiovale; SVD: cerebral small vessel disease; OCTA: optical coherence tomography angiography.

*Note*: For SVD markers, data were displayed as median [IQR] and number (frequency).

**FIGURE 2:**
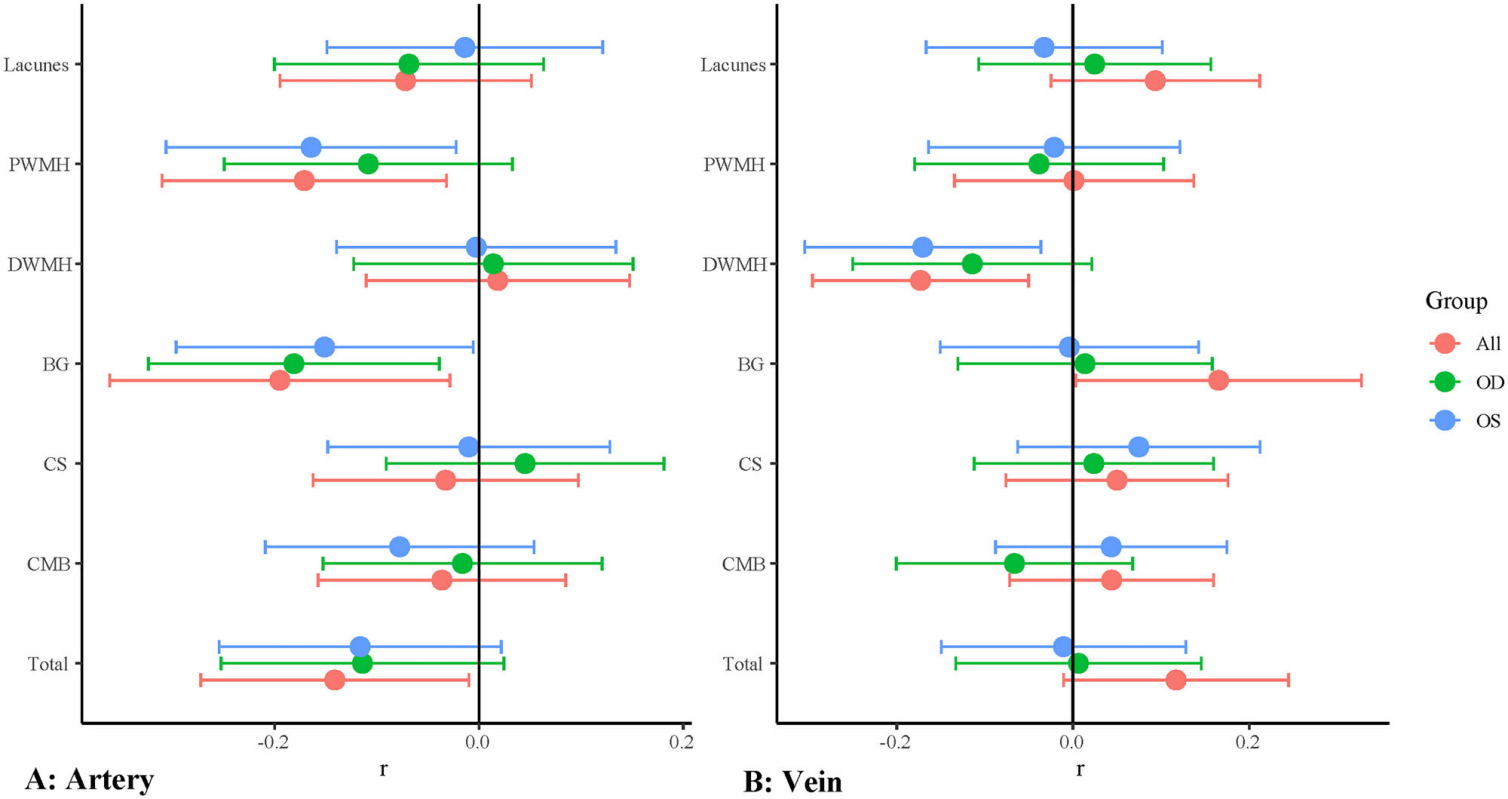
**Adjusted models showing the association between SVD markers and density of retinal artery/vein**. Generalized estimating equations were used for per person (all in figure) while in per eyes (OD/OS in figure), linear regressions were conducted. *r*: standardized regression coefficient.

**FIGURE 3:**
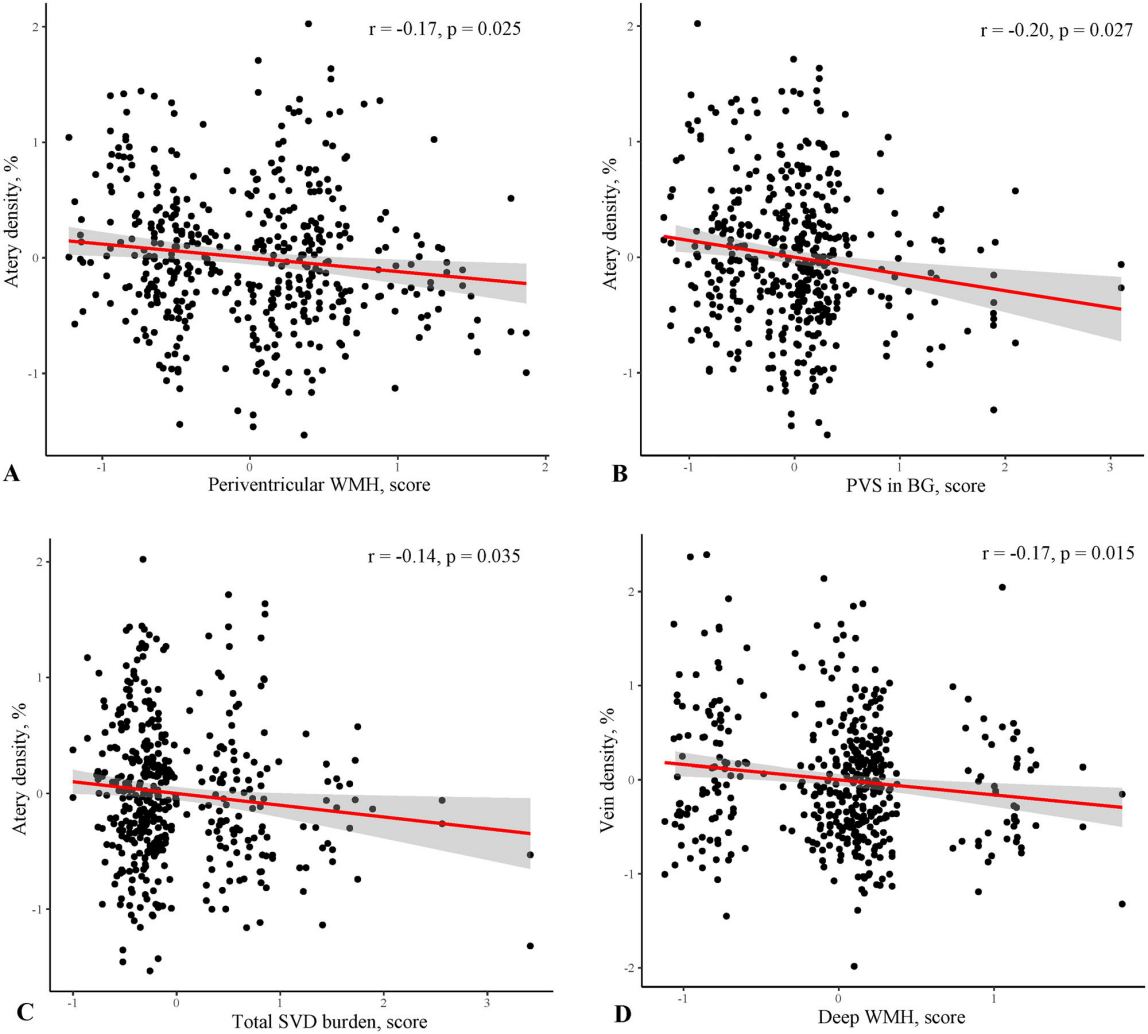
**Partial correlation between artery/vein density and SVD markers. (**A) Artery density and periventricular WMH. (B) Artery density and PVS in BG. (C) Artery density and total SVD burden. (D) Vein density and deep WMH. Added variable plot were used; the axis were the residuals when these variables were regressed on the covariates.

As shown in Figure S2 in our unadjusted analyses, lower arteriolar density was associated with periventricular WMH and PVS in the basal ganglia while venule density was associated with deep WMH (all *p* < 0.05). After adjusting for covariates as shown in Figures 2 and 3 and Table S1, lower arteriolar density was associated with higher periventricular WMH (*r* = −0.17, 95% CI −0.31 to −0.03, *p* = 0.025) and PVS in the basal ganglia (*r* = −0.20, 95% CI −0.36 to −0.03, *p* = 0.027) while lower venular density was associated with higher deep WMH burden (*r* = −0.17, 95% CI −0.30 to −0.05, *p* = 0.015). Importantly, lower arteriolar density was associated with increased SVD burden (*r* = −0.14, 95% CI −0.27 to −0.01, *p* = 0.035). Besides, less arteriolar complex branching was associated with increased periventricular WMH (*r* = −0.18, 95% CI −0.32 to −0.04, *p* = 0.020). Less venular complex branching was associated with higher deep WMH (*r* = −0.12, 95% CI −0.24 to −0.01, *p* = 0.041). Table S2 and Figure S3 show the association between complex branching of retinal artery/vein and SVD markers.

## 4 Discussion

This study explored the association between a range of retinal microvascular network metrics on OCTA and SVD markers in a study cohort who were dementia and stroke free. We found that lower retinal arteriolar density was associated with higher periventricular WMH burden and PVS in the basal ganglia while lower retinal venular density was associated with higher deep WMH burden. Furthermore, lower retinal arteriolar density was associated with higher SVD burden. We also showed that less arteriolar branching complexity was associated with higher periventricular WMH and PVS in the basal ganglia. To the best of our knowledge, this is the first study to report such findings which may be of significance as they demonstrate a potential relationship of retinal arteriolar and venular changes with SVD markers.

Retinal arterioles are the main resistance vessels of the retina, and consequently, play a key role in regulating retinal hemodynamics (Curtis et al. 2018) Accumulating studies have shown that changes in retinal arterioles are associated with hypertension and cardiovascular disorders (Wong et al. 2001; Wong et al. 2003). On the other hand, changes in the retinal venules (specifically widening of the retinal venules) are considered as indicators of cerebrovascular disorders such as ischemic stroke (Doubal et al. 2009; Doubal, Hokke et al. 2009; Lindley et al. 2009; Yatsuya et al. 2010).

Older adults with cerebral SVD often have WMH on brain MRI. Previous studies (Ashimatey et al. 2020) showed that reduced retinal density on OCTA was associated with increased an Fazekas score in cognitively normal adults. Here we showed that lower arteriolar and venule densities correlated with high periventricular and deep WMH (by visual score, Fazekas scale) in older adults respectively. Periventricular WMH has been shown to have a strong association with cardiovascular risk factors (Sudre et al. 2018), as it is believed to be particularly susceptible to decreases in blood flow due to its location at the arterial border zone (Spilt et al. 2006). Similarly, retinal imaging reports have shown arterial changes are associated with cardiovascular risk factors such as hypertension (Kawasaki et al. 2009) and diabetes mellitus (Wong et al. 2005). In these studies, retinal arterioles narrowed and thickened after longstanding hypertension. As both periventricular WMH and arteriolar changes of the retina are strongly linked with cardiovascular risk factors, it is plausible to suggest that lower retinal arteriolar densities are linked with increased periventricular WMH burden assessed by the Fazekas scale. Deep WMH is associated with neuropathological changes that result from ischemia and is closely related to SVD (Garnier‐Crussard et al. 2023). It is hypothesized that retinal venular dilation (reduced venular density) occurs in response to retinal hypoxia (Cheng et al. 2016). Previous retinal imaging reports (Cheng et al. 2016; Klein et al. 2004) demonstrated venular dilatation as one of the early changes in retinal hypoxia. Here, we showed that lower retinal venular density was associated with higher deep WMH burden in older adults. Based on these observations, a possible explanation might be that reduced venular density is a general marker of diffuse retinal ischemia and by proxy cerebral ischemia leading to increased susceptibility of brain tissue to the development of deep WMH.

PVS is one of several markers of vascular dysfunction in the brain that occurs early in SVD development (Duering et al. 2023). Previous studies (Ballerini et al. 2020; Mutlu et al. 2016) showed a correlation between increasing PVS volumes and decreasing fractal dimension of larger retinal arterioles in fundus photography. Here we showed strong associations between decreased arteriolar density and increased PVS in the basal ganglia. This association remained after adjusting for covariates. PVS in the basal ganglia is mostly related to vascular risk factors such as hypertension (Ramaswamy et al. 2022). A nearly vertical bifurcation angle in the perforating arteries of the basal ganglia makes it susceptible to high blood pressure. As both PVS in the basal ganglia and retinal arterioles are linked with hypertension, reduced arteriolar densities may be linked with higher PVS in the basal ganglia.

Cerebral small vessel disease (SVD), the accumulation of the 4 MRI features, is an intrinsic disorder of the small perforating arterioles in the brain (Duering et al. 2023; Staals et al. 2014). Here we showed that lower arteriolar density is associated with increased SVD burden suggesting that reduced arteriolar damage may be linked with deterioration of SVD burden in older adults.

We conducted a unilateral model to assess the association between OCTA metrics and SVD markers based on images from a single eye. A unilateral model is essential because the retinal image of one eye might not be assessable due to macular pathologies such as cataracts; these pathologies are prevalent in older adults. Based on single‐eye OCTA images, the unilateral model shows that retinal microvascular changes are associated with SVD markers.

In light of recent research, it appears that a reliable and early indicator of the disease can be used to initiate effective treatment during the quiescent phase. As SVD alterations accompany retinal microvascular changes, the concept of cerebral SVD manifestation in the retina has evolved in recent years. Researchers have reported correlations between retinal microvasculature and cerebral SVD markers using OCTA technology (García‐Sánchez et al. 2024; Tao et al. 2022; Wiseman et al. 2023). Here we showed retinal arterioles and venules imaged on OCTA correlated with WMH and PVS‐BG in older adults. This emphasizes the importance of retinal imaging via OCTA for assessing the subtle, yet discrete changes associated with aging, a risk factor for SVD. The success of this study was attributed to several factors, including the large sample size of elderly adults, the analysis of both eyes of each participant, the cerebral imaging protocol used to generate the brain markers described in SVD, and the adjustment for risk factors.

We would like to acknowledge some limitations in our study. A major limitation is the cross‐sectional design of our study. A longitudinal study is needed to track the progression of retinal and cerebral changes over time which may provide insights into the causal relationships and predictive value of OCTA measurements. The exclusion of participants from the study may cause a selection bias and underestimation of the observed associations; participants with cerebral disorders on MR imaging and retinal abnormalities were excluded. But neurologic and ophthalmic disorders cannot be avoided in older adults; future studies with larger sample sizes can include participants with these disorders and controlled for in their statistics. This study was conducted in a local community in China, which may limit the generalizability of the findings. The findings in current study should be validated in other races and regions. The assessment of SVD were limited by visual rating. Developments in neuroimaging software have established several semiautomated methods for estimating SVD markers such as WMH and PVS volume, which may be the current analytic standards. Further studies are required to explore the association between quantitative SVD markers and OCTA measurements.

In conclusion, we showed that retinal microvascular changes identified on OCTA were associated with SVD markers. Since OCTA measures are relatively easy to tolerate and economical, they hold promise as noninvasive biomarkers of cerebral SVD. The predictive power of OCTA measurements for SVD progression in older adults and monitoring treatment response require longitudinal studies.

## Author Contributions

**Le Cao**: conceptualization, investigation, writing–original draft, methodology, visualization, writing–review and editing, formal analysis. **Hang Wang**: conceptualization, investigation, writing–original draft, methodology, visualization, writing–review and editing, software. **Jinkui Hao**: investigation, methodology, visualization, writing–review and editing, formal analysis, software, conceptualization. **William Robert Kwapong**: conceptualization, investigation, writing–review and editing. **Zhouwei Xiong**: visualization, software. **Ruilin Wang**: investigation, supervision. **Chen Ye**: investigation. **Yitian Zhao**: writing–review and editing, software, supervision, methodology. **Bo Wu**: conceptualization, funding acquisition, writing–review and editing, visualization, methodology, supervision. **Wendan Tao**: conceptualization, funding acquisition, writing–review and editing, software, supervision.

## Ethics Statement

The West China Hospital of Sichuan University Ethics Committee approved the study (Ethics number 2020[922]).

### Peer Review

The peer review history for this article is available at https://publons.com/publon/10.1002/brb3.70342.

## Supporting information



Figure S1: Distributions of SVD markers.

Figure S2. Unadjusted univariate models for association between density of retinal artery/vein and SVD marker.

Figure S3: Adjusted models showing the association between SVD markers and fractal dimension of retinal artery/vein.

Table S1: Association between density of retinal artery/vein and SVD marker, adjusted for age, sex, hypertension, diabetes mellitus, hyperlipidemia, and smoking.
Table S2: Association between fractal dimension of retinal artery/vein and SVD marker, adjusted for age, sex, hypertension, diabetes mellitus, hyperlipidemia, and smoking.

## Data Availability

The data that support the findings of this study are available on request from the corresponding author.
