# Supplementary figures and images for: Lower Retinal Arteriolar Density Is Associated With Higher Cerebral Small Vessel Disease Burden: An Optical Coherence Tomography Angiography Study
Source: Brain Behav. 2025 Feb 17;15(2):e70342. doi: 10.1002/brb3.70342 (PMC11830995; doi:10.1002/brb3.70342)

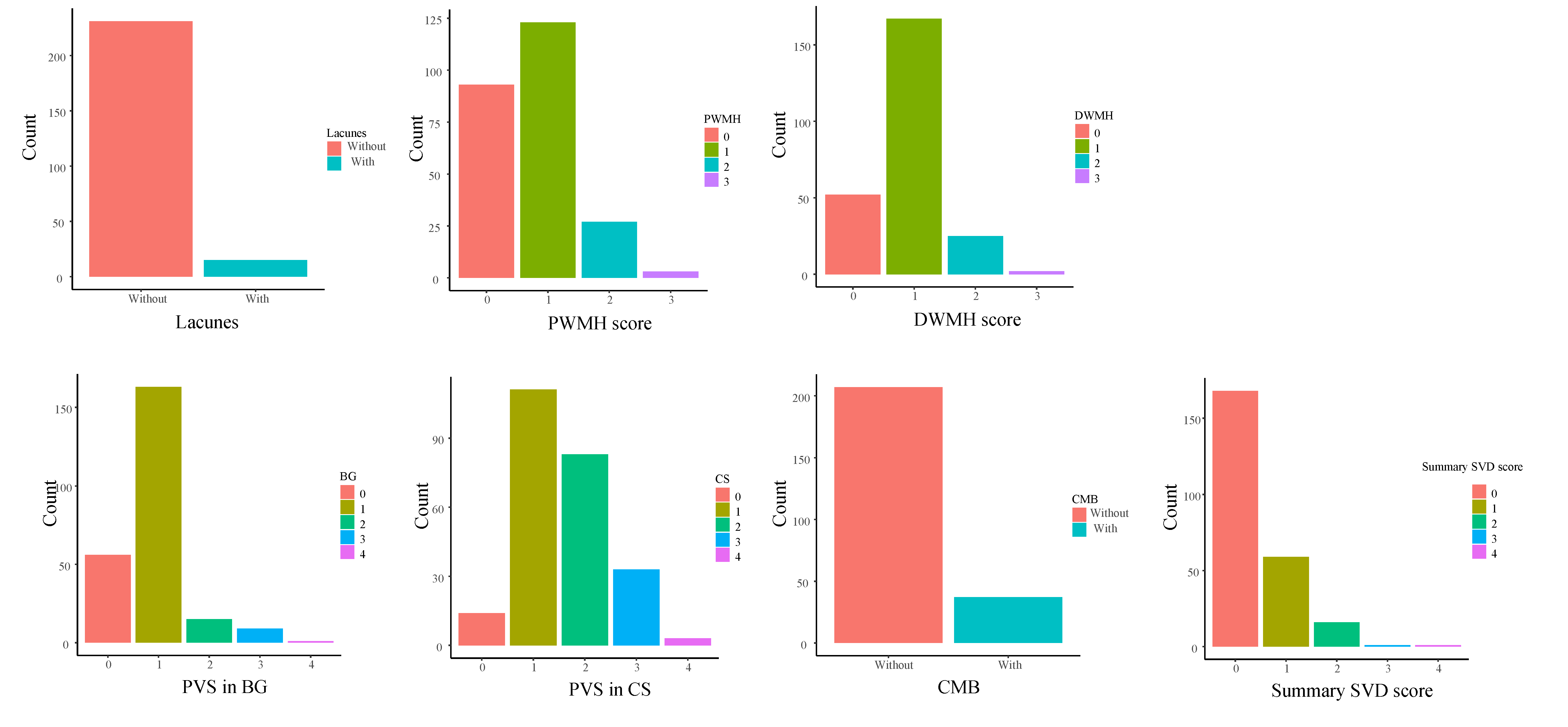

Supplement: Supplementary file 1 — Figure S1: Distributions of SVD markers. [file BRB3-15-e70342-s003.tiff]

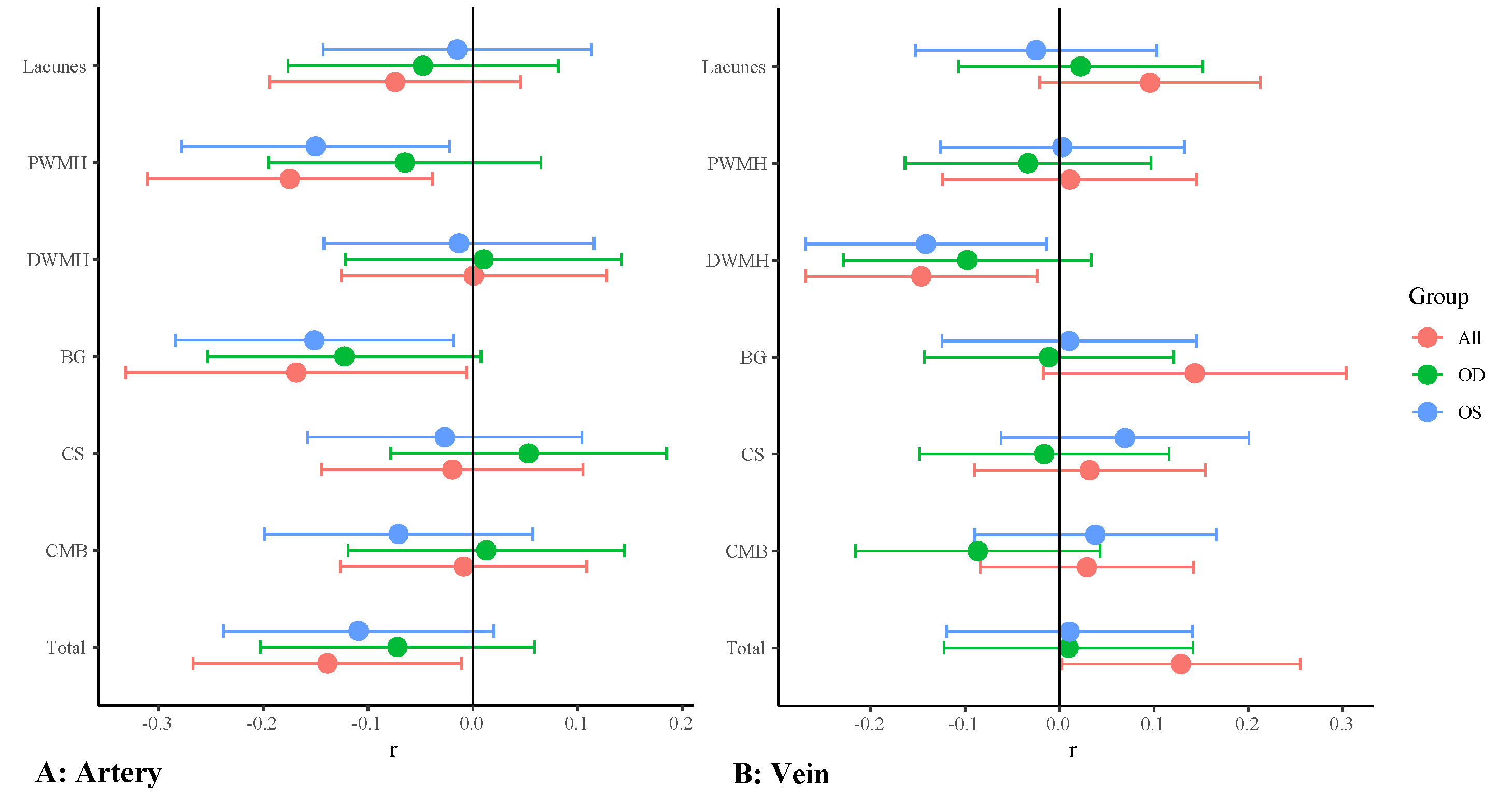

Supplement: Supplementary file 2 — Figure S2. Unadjusted univariate models for association between density of retinal artery/vein and SVD marker. [file BRB3-15-e70342-s004.tif]

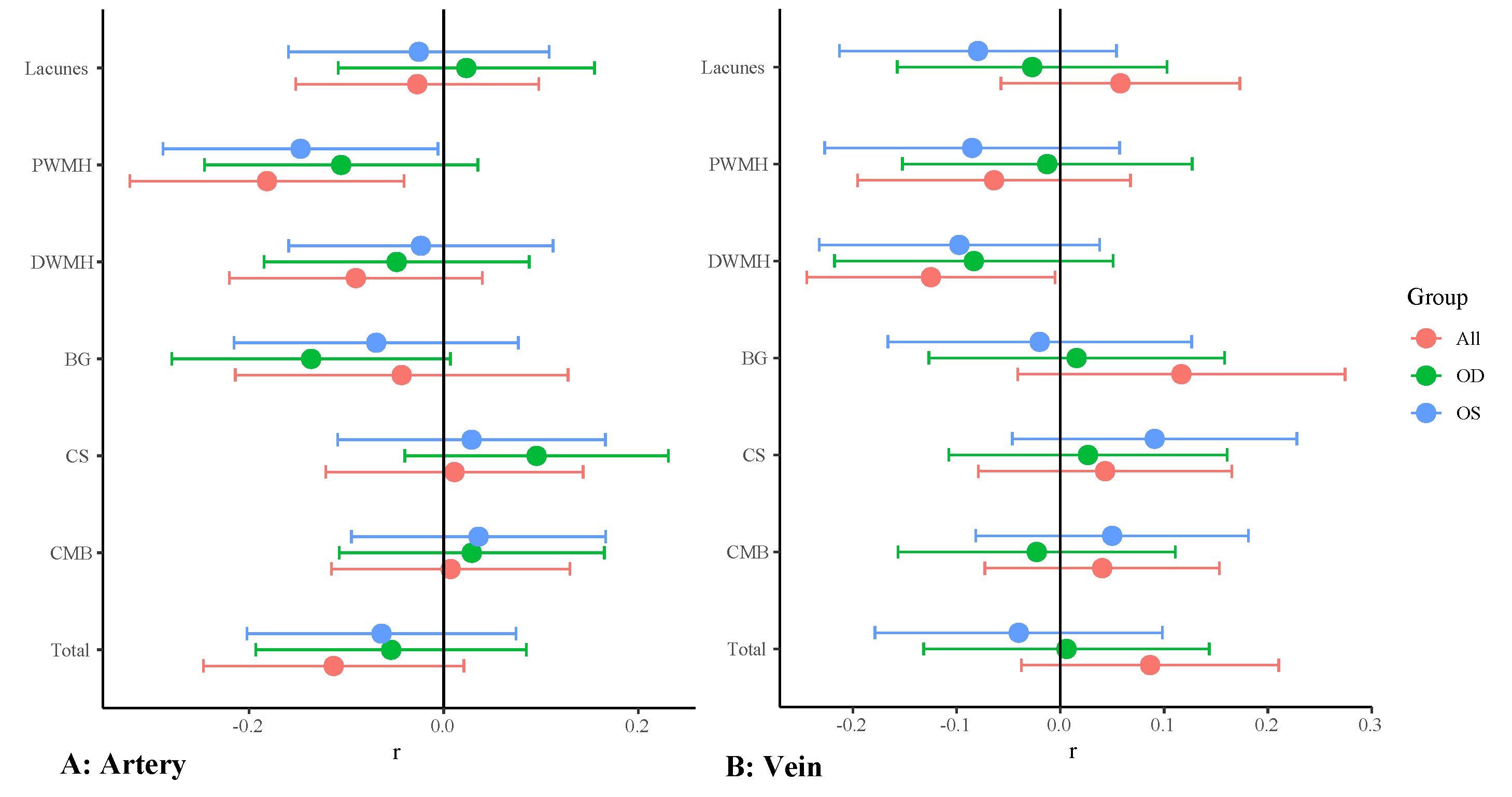

Supplement: Supplementary file 3 — Figure S3: Adjusted models showing the association between SVD markers and fractal dimension of retinal artery/vein. [file BRB3-15-e70342-s001.tif]
