# Supplementary material for: Lower Retinal Arteriolar Density Is Associated With Higher Cerebral Small Vessel Disease Burden: An Optical Coherence Tomography Angiography Study
Source: Brain Behav. 2025 Feb 17;15(2):e70342. doi: 10.1002/brb3.70342 (PMC11830995; doi:10.1002/brb3.70342)
Supplement: Supplementary file 4 — Table S1: Association between density of retinal artery/vein and SVD marker, adjusted for age, sex, hypertension, diabetes mellitus, hyperlipidemia, and smoking. Table S2: Association between fractal dimension of retinal artery/vein and SVD marker, adjusted for age, sex, hypertension, diabetes mellitus, hyperlipidemia, and smoking. [file BRB3-15-e70342-s002.docx]

Supplementary Table 1: Association between density of retinal artery/vein and SVD marker, adjusted for age, sex, hypertension, diabetes mellitus, hyperlipidemia, and smoking.

|  | Artery | | | Vein | | |
| --- | --- | --- | --- | --- | --- | --- |
|  | r | 95%CI | p | r | 95%CI | p |
| All |  |  |  |  |  |  |
| Lacunes | -0.07 | -0.2~0.05 | 0.196 | 0.09 | -0.03~0.21 | 0.306 |
| PWMH | -0.17 | -0.31~-0.03 | 0.025 | 0.00 | -0.13~0.14 | 0.988 |
| DWMH | 0.02 | -0.11~0.15 | 0.805 | -0.17 | -0.30~-0.05 | 0.015 |
| BG | -0.20 | -0.36~-0.03 | 0.027 | 0.17 | 0.00~0.33 | 0.144 |
| CS | -0.03 | -0.16~0.10 | 0.655 | 0.05 | -0.08~0.18 | 0.501 |
| CMB | -0.04 | -0.16~0.09 | 0.675 | 0.04 | -0.07~0.16 | 0.527 |
| Total | -0.14 | -0.27~-0.01 | 0.035 | 0.12 | -0.01~0.24 | 0.278 |
| OD |  |  |  |  |  |  |
| Lacunes | -0.07 | -0.2~0.06 | 0.309 | 0.02 | -0.11~0.16 | 0.715 |
| PWMH | -0.11 | -0.25~0.03 | 0.134 | -0.04 | -0.18~0.1 | 0.594 |
| DWMH | 0.01 | -0.12~0.15 | 0.840 | -0.11 | -0.25~0.02 | 0.100 |
| BG | -0.18 | -0.32~-0.04 | 0.013 | 0.01 | -0.13~0.16 | 0.854 |
| CS | 0.05 | -0.09~0.18 | 0.516 | 0.02 | -0.11~0.16 | 0.733 |
| CMB | -0.02 | -0.15~0.12 | 0.817 | -0.07 | -0.2~0.07 | 0.334 |
| Total | -0.11 | -0.25~0.02 | 0.108 | 0.01 | -0.13~0.15 | 0.931 |
| OS |  |  |  |  |  |  |
| Lacunes | -0.01 | -0.15~0.12 | 0.841 | -0.03 | -0.17~0.1 | 0.632 |
| PWMH | -0.16 | -0.31~-0.02 | 0.024 | -0.02 | -0.16~0.12 | 0.769 |
| DWMH | 0.00 | -0.14~0.13 | 0.970 | -0.17 | -0.3~-0.04 | 0.013 |
| BG | -0.15 | -0.3~-0.01 | 0.043 | 0.00 | -0.15~0.14 | 0.956 |
| CS | -0.01 | -0.15~0.13 | 0.888 | 0.07 | -0.06~0.21 | 0.288 |
| CMB | -0.08 | -0.21~0.05 | 0.249 | 0.04 | -0.09~0.17 | 0.519 |
| Total | -0.12 | -0.25~0.02 | 0.100 | -0.01 | -0.15~0.13 | 0.880 |

Footnotes: PWMH: periventricular white matter hyperintensity; DWMH: deep white matter hyperintensity; BG: perivascular spaces in basal ganglia; CS: periventricular spaces in centrum semiovale; CMB: cerebral microbleeds; SVD: cerebral small vessel disease. r: standardized regression coefficient; p values were calculated by GEE.

Supplementary Table 2: Association between fractal dimension of retinal artery/vein and SVD marker, adjusted for age, sex, hypertension, diabetes mellitus, hyperlipidemia, and smoking.

|  | Artery | | | Vein | | |
| --- | --- | --- | --- | --- | --- | --- |
|  | r | 95%CI | p | r | 95%CI | p |
| All |  |  |  |  |  |  |
| Lacunes | -0.03 | -0.15~0.1 | 0.668 | 0.06 | -0.06~0.17 | 0.423 |
| PWMH | -0.18 | -0.32~-0.04 | 0.020 | -0.06 | -0.2~0.07 | 0.364 |
| DWMH | -0.09 | -0.22~0.04 | 0.223 | -0.12 | -0.24~-0.01 | 0.041 |
| BG | -0.04 | -0.21~0.13 | 0.627 | 0.12 | -0.04~0.27 | 0.252 |
| CS | 0.01 | -0.12~0.14 | 0.886 | 0.04 | -0.08~0.17 | 0.555 |
| CMB | 0.01 | -0.12~0.13 | 0.937 | 0.04 | -0.07~0.15 | 0.514 |
| Total | -0.11 | -0.25~0.02 | 0.137 | 0.09 | -0.04~0.21 | 0.314 |
| OD |  |  |  |  |  |  |
| Lacunes | 0.02 | -0.11~0.15 | 0.730 | -0.03 | -0.16~0.1 | 0.683 |
| PWMH | -0.11 | -0.25~0.04 | 0.143 | -0.01 | -0.15~0.13 | 0.859 |
| DWMH | -0.05 | -0.18~0.09 | 0.487 | -0.08 | -0.22~0.05 | 0.225 |
| BG | -0.14 | -0.28~0.01 | 0.063 | 0.02 | -0.13~0.16 | 0.829 |
| CS | 0.10 | -0.04~0.23 | 0.169 | 0.03 | -0.11~0.16 | 0.698 |
| CMB | 0.03 | -0.11~0.16 | 0.680 | -0.02 | -0.16~0.11 | 0.739 |
| Total | -0.05 | -0.19~0.08 | 0.447 | 0.01 | -0.13~0.14 | 0.933 |
| OS |  |  |  |  |  |  |
| Lacunes | -0.03 | -0.16~0.11 | 0.708 | -0.08 | -0.21~0.05 | 0.245 |
| PWMH | -0.15 | -0.29~-0.01 | 0.042 | -0.08 | -0.23~0.06 | 0.242 |
| DWMH | -0.02 | -0.16~0.11 | 0.734 | -0.10 | -0.23~0.04 | 0.160 |
| BG | -0.07 | -0.22~0.08 | 0.353 | -0.02 | -0.17~0.13 | 0.790 |
| CS | 0.03 | -0.11~0.17 | 0.684 | 0.09 | -0.05~0.23 | 0.195 |
| CMB | 0.04 | -0.1~0.17 | 0.593 | 0.05 | -0.08~0.18 | 0.457 |
| Total | -0.06 | -0.2~0.07 | 0.365 | -0.04 | -0.18~0.1 | 0.571 |

Footnotes: PWMH: periventricular white matter hyperintensity; DWMH: deep white matter hyperintensity; BG: perivascular spaces in basal ganglia; CS: periventricular spaces in centrum semiovale; CMB: cerebral microbleeds; SVD: cerebral small vessel disease. r: standardized regression coefficient; p values were calculated by GEE.
